# Differences in the expression of SSTR1–5 in meningiomas and its therapeutic potential

**DOI:** 10.1007/s10143-021-01552-y

**Published:** 2021-04-26

**Authors:** Felix Behling, Christina Fodi, Marco Skardelly, Mirjam Renovanz, Salvador Castaneda, Ghazaleh Tabatabai, Jürgen Honegger, Marcos Tatagiba, Jens Schittenhelm

**Affiliations:** 1 Department of Neurosurgery, University Hospital Tübingen, Eberhard Karls University, Hoppe-Seyler Street 3, Tübingen, Germany; 2 Center for CNS Tumors, Comprehensive Cancer Center Tübingen-Stuttgart, University Hospital Tübingen, Eberhard Karls University, Tübingen, Germany; 3 German Cancer Consortium (DKTK), DKFZ Partner Site Tübingen, Tübingen, Germany; 4 Interdisciplinary Division of Neuro-Oncology, University Hospital Tübingen, Eberhard Karls University, Tübingen, Germany; 5 Department of Nuclear Medicine, University Hospital Tübingen, Eberhard Karls University, Tübingen, Germany; 6 Department of Neurology, University Hospital Tübingen, Eberhard Karls University, Tübingen, Germany; 7 Hertie Institute for Clinical Brain Research, Tübingen, Germany; 8 Department of Neuropathology, University Hospital Tübingen, Eberhard Karls University, Tübingen, Germany

**Keywords:** Meningioma, Somatostatin receptor, Peptide receptor radionuclide therapy (PRRT), Neurofibromatosis, Immunohistochemistry, Tissue microarray

## Abstract

Beyond microsurgical resection and radiation therapy, there are currently no established treatment alternatives for meningioma patients. In selected cases, peptide radio receptor therapy (PRRT) can be implemented. For this purpose, a radionuclide is bound to a substance targeting specific receptors in meningiomas. One of them is somatostatin receptor 2, which can be found in most meningiomas. However, other somatostatin receptors (SSTR) exist, but their expressions have only been described in small case series. In this study, we analyzed the expression of SSTR1, 2A, 3, 4, and 5 in a large cohort of meningiomas in order to enable further refinement of this innovative treatment option. Overall, 726 tumor samples were processed into tissue microarrays and stained for SSTR1, 2A, 3, 4, and 5 immunohistochemically. Microscopic evaluation was done with an established semiquantitative score regarding percentual quantification and staining intensity, and results were correlated with clinical data. There was a significant lower rate of SSTR1 expression in meningiomas of male patients. Older age was associated with higher expression of SSTR1, 2A, and 5 and lower scores for SSTR3 and 4. Tumors treated with radiotherapy before resection showed lower rates of SSTR1 and 5 expression, while recurrent meningiomas had lower SSTR1 scores. Tumor tissue from patients suffering from neurofibromatosis type 2 had lower expression scores for SSTR1, 2, and 5. For SSTR3 and 4, NF2 patients showed higher scores than sporadic tumors. Spinal meningiomas had higher scores for SSTR1, 4, and 5 compared tumor location of the skull base and convexity/falx. Overall, higher WHO grade was associated with lower SSTR scores. While all SSTRs were expressed, there are marked differences of SSTR expression between meningioma subgroups. This has the potential to drive the development of more selective PRRT substances with higher treatment efficacy.

**Supplementary Information:**

The online version contains supplementary material available at 10.1007/s10143-021-01552-y.

## Introduction

Meningioma is the most common benign intracranial neoplasm. It represents 36% of all central nervous system tumors with only 1.3% showing malignant features [29]. If a meningioma causes symptoms, it shows significant growth or has reached a critical size; treatment is required [15]. In most cases patients, can be cured by radical surgical resection [35]. Radiation is also a primary treatment option in selected cases and plays a significant role in the treatment of recurring or higher graded meningiomas [6] as well as meningiomas in difficult locations in elderly patients [15]. Besides several clinical studies, no medical treatment option was able to achieve a usable antitumor efficacy [5, 21]. Peptide receptor radionuclide therapy (PRRT), however, is a promising treatment option first established in gastroenteropancreatic neuroendocrine tumors (GEP-NET) [24, 40]. Six different somatostatin receptor types (SSTR1, SSTR2A, SSTR2B, SSTR3, SSTR4, and SSTR5) have been detected in numerous different human tissues [30] as well as several tumor types [25, 39]. Tumors can express more than one SSTR. Such tumor tissue that expresses somatostatin receptors can be approached with radionuclide labeled somatostatin analogs [12, 41]. By itself, somatostatin is known to have antiproliferative and anti-angiogenic effects [31, 34]. Coupled with a radionuclide, somatostatin receptor expressing tumor tissue can be specifically targeted with no significant damage to normal tissue. This treatment concept has also been investigated in other tumor tissues [9], and clinical efficacy of PRRT in selected recurrent meningioma cases has been reported [4, 23, 27, 37].

Some studies have analyzed the distribution of somatostatin receptors in meningiomas, especially SSTR2A. However, the patient cohorts were small, mainly consisted of WHO grade I meningiomas, and the tumor grading in these studies deviates from the current WHO classification of central nervous system tumors in which CNS infiltration in meningiomas significantly influences the grading [2, 3, 11, 26, 31, 36, 38]. But especially patients with WHO grade II and III meningiomas and neurofibromatosis type 2 (NF2), who frequently develop many intracranial meningiomas [1], are subjects that need to be further assessed regarding this targeted therapy option. Therefore, we analyzed the distribution of 5 somatostatin receptors (SSTR1, SSTR2A, SSTR3, SSTR4, SSTR5) in a large meningioma cohort including patients suffering from NF2 and higher grade meningiomas.

## Materials and methods

### Patient cohort

Between January 2013 and March 2017, a total of 632 meningiomas were resected in the authors’ institution. Additionally, 94 meningiomas of WHO grade II and III as well as meningiomas from patients suffering from neurofibromatosis type 2 that were treated between July 2003 and March 2017 were included in order to have larger subgroups of these less common patients that are potential candidates for PRRT. Overall, paraffin-embedded tumor tissue samples of 726 meningiomas were available and suitable for tissue microarray construction. Furthermore, the following clinical data was collected: age, gender, histopathological diagnosis (2016 WHO classification), presence of NF2, prior radiotherapy, primary or recurrent tumor, and tumor location.

### Tissue microarray and immunohistochemistry

All meningiomas were histopathologically graded using the 2016 WHO classification for central nervous system tumors [26]. Provided by the Department of Neuropathology, paraffin-embedded tumor tissue samples were used for the construction of tissue microarrays. Representative areas for 1-mm tissue cylinder extraction were marked after histological evaluation of the corresponding hematoxylin eosin stain. A conventional tissue microarrayer was used (Beecher Instruments, Sun Prairie, Wisconsin, USA) to extract 2 sample cylinders from different tumor regions if enough representative tumor tissue was available, which was the case for most tumors. Via microtomy, 4-μm slices from the acceptor block were prepared, and after drying at 65° Celsius for 15 min, immunohistochemical staining was performed with a Ventana BenchMark immunostainer (Ventana Medical Systems, Tucson, Arizona, USA). The OptiView method was applied. For SSTR1, pretreatment was done with protease for 4 min, for SSTR2A with CC2 for 32 min, and for SSTR3–5 with CC1 for 32 min. Primary antibodies were administered at 37 °C for 40 min for SSTR1, for 120 min for SSTR2A, and for 32 min for SSTR3–5. The following dilutions were used: SSTR1, 1:3000 (Gramsch, Schwabhausen, Germany); SSTR2A, 1:500 (Dianova, Hamburg, Germany); SSTR3, 1:1000 (Abcam, Cambridge, UK); SSTR4, 1:1000 (Gentex, Zeeland, USA); and SSTR5, 1:100 (Abcam, Cambridge, UK). Antibodies were validated with pancreatic tissue slides which were used as separate controls.

### Microscopic assessment and statistical methods

To determine the expression of SSTR1–5 with regard to intensity and quantity, an intensity distribution score was applied as described by Barresi et al. [3] (Table 1) as meningiomas exhibit a similar receptor density as gastroenteropancreatic neuroendocrine tumors [30]. Microscopic assessment was done by two investigators, and in difficult cases, a rating consensus was reached between both investigators. Statistical analysis was done with JMP® Statistical Discovery Software, version 15.1.0 (Cary, NC: SAS Institute Inc.; 1989). The one-way ANOVA test was applied with a significance level of α < 0.05. A classification and regression tree (CART) analysis was done for the identification of age cutoffs regarding maximum differences in somatostatin expression for all 5 markers.

**Table 1:** Grading of immunohistopositivity according to Barresi et al. [3]

Intensity distribution score (ID) = IS × ASP	0–12
Immunostaining intensity (IS)	
Negative	0
Weak	1
Moderate	2
Strong	3
Area of staining positivity (ASP)	
< 5%	0
5–25%	1
26–50%	2
51–75%	3
76–100%	4

## Results

### Cohort characteristics

Overall, the immunohistochemical results together with clinical data of 726 meningiomas were analyzed. Sixty-nine percent of the meningiomas were diagnosed in female patients (502/726), while 31% were male (224/726). The mean age was 56.7 years ranging from 8.3 to 89.9 years. With 85%, the majority of cases were primary meningiomas (613/726), whereas 15% were surgically resected for tumor recurrence (113/726). Eight percent of cases received prior radiotherapy (61/726); 90% of these cases were recurrent meningiomas (55/61). Seventy-three tumors were resected from NF2 patients (10%). The majority of tumors were skull base meningiomas (52%, 375/726), while 39% were localized at the convexity or falx (282/726) and 10% along the spine (69/726). Details are displayed in Table 2. According to the WHO classification of 2016, 81% were grade I meningiomas, 16% grade II, and 3% grade III. The distribution of histological subgroups is shown in Table 3.

**Table 2:** Cohort characteristics and immunohistochemical distribution of somatostatin receptor expression (ANOVA)

		SSTR1		SSTR2A		SSTR3		SSTR4		SSTR5	
	*N* (%)	Mean (95%CI)	*p* Value	Mean (95%CI)	*p* Value	Mean (95%CI)	*p* Value	Mean (95%CI)	*p* Value	Mean (95%CI)	*p* Value
Gender											
Female	502 (69.1)	6.7 (6.4–6.9)	0.0269*	5.8 (5.5–6.0)	0.2076	2.2 (2.0–2.4)	0.4467	2.8 (2.7–3.0)	0.0806	4.8 (4.7–5.0)	0.9765
Male	224 (30.9)	6.2 (5.8–6.5)		6.0 (5.7–6.4)		2.3 (2.0–2.6)		2.6 (2.4–2.8)		4.8 (4.6–5.1)	
Age (cutoff according to CART)		41.91 years		34.47 years		72.45 years		42.98 years		45.77 years	
≥		6.7 (6.5–6.9)	< .0001*	5.9 (5.7–6.1)	0.0011*	1.4 (1.0–1.8)	< .0001*	2.6 (2.5–2.8)	0.0005*	5.0 (4.8–5.1)	0.0002*
<		5.5 (5.0–6.0)		4.7 (4.0–5.4)		2.4 (2.2–3.6)		3.2 (2.9–3.5)		4.3 (4.1–4.6)	
Recurrence											
Primary	613 (84.4)	6.7 (6.5–6.9)	< .0001*	5.8 (5.6–6.0)	0.3696	2.2 (2.0–2.3)	0.3610	2.8 (2.6–2.9)	0.2436	4.9 (4.7–5.0)	0.1503
Recurrence	113 (15.6)	5.5 (5.0–6.0)		6.1 (5.6–6.5)		2.4 (2.0–2.8)		2.6 (2.3–2.9)		4.6 (4.2–4.9)	
Prior radiation											
Yes	61 (8.4)	5.4 (4.7–6.1)	0.0007*	6.1 (5.4–6.8)	0.4835	2.1 (1.5–2.6)	0.6414	2.4 (2.0–2.9)	0.1291	4.3 (3.9–4.8)	0.0340*
No	665 (91.6)	6.6 (6.4–6.8)		5.8 (5.6–6.0)		2.2 (2.1–2.4)		2.8 (2.7–2.9)		4.9 (4.7–5.0)	
NF2											
Yes	73 (10.1)	4.6 (4.0–5.2)	< .0001*	5.0 (4.4–5.6)	0.0049*	3.8 (3.3–4.3)	< .0001*	3.6 (3.3–4.0)	< .0001*	3.9 (3.5–4.3)	< .0001*
No	653 (90.0)	6.7 (6.5–6.9)		6.0 (5.8–6.2)		2.0 (1.9–2.2)		2.6 (2.5–2.8)		5.0 (4.8–5.1)	
Localization											
Convexity/Falx	282 (38.8)	5.8 (5.5–6.2)	< .0001*	5.6 (5.3–5.9)	0.0003*	1.7 (1.5–2.0)	< .0001*	2.6 (2.4–2.8)	0.0062*	4.5 (4.3–4.7)	< .0001*
Skull base	375 (51.7)	6.7 (6.5–7.0)		6.2 (5.9–6.5)		2.6 (2.4–2.9)		2.8 (2.6–2.9)		4.9 (4.7–5.1)	
Spinal	69 (9.5)	8.1 (7.5–8.8)		4.9 (4.3–5.6)		1.7 (1.2–2.2)		3.3 (2.9–3.7)		5.7 (5.2–6.1)	
2016 WHO classification											
I	585 (80.6)	6.8 (6.6–7.0)	< .0001*	5.8 (5.5–6.0)	0.0160*	2.2 (2.0–2.4)	0.8841	2.9 (2.7–3.0)	0.0011*	4.9 (4.7–5.0)	0.0003*
II	118 (16.3)	5.6 (5.1–6.1)		6.5 (6.0–7.0)		2.2 (1.8–2.6)		2.4 (2.1–2.7)		4.8 (4.4–5.1)	
III	23 (3.2)	4.6 (3.5–5.8)		5.5 (4.4–6.5)		2.0 (1.1–2.9)		2.0 (1.3–2.6)		3.3 (2.5–4.1)	

Abbreviations: *SSTR* somatostatin receptor, *CI* confidence interval, *CART* classification and regression tree, *NF2* neurofibromatosis type 2, *WHO* World Health Organization, *ANOVA* analysis of variance; asterisk (*) presents statistically significant results

**Table 3:** Somatostatin receptor expression according to histology

	SSTR1		SSTR2A		SSTR3		SSTR4		SSTR5	
	*N*	Mean (95%CI)	*N*	Mean (95%CI)	*N*	Mean (95%CI)	*N*	Mean (95%CI)	*N*	Mean (95%CI)
WHO I										
Angiomatous	16	7.5 (6.3–8.8)	16	7.9 (6.8–9.0)	16	2.0 (0.9–3.0)	16	3.6 (2.8–4.4)	16	6.9 (6.0–7.8)
Fibroblastic	52	6.2 (5.5–6.9)	54	4.1 (3.4–4.7)	52	1.5 (1.0–2.1)	53	2.7 (2.3–3.1)	54	4.1 (3.6–4.6)
Lymphocyte rich	1	8.0	1	4.0	1	1.0	1	2.5	1	4.0
Meningothelial	334	6.8 (6.5–7.1)	334	5.9 (5.6–6.1)	336	2.3 (2.1–2.5)	339	3.0 (2.8–3.1)	340	5.1 (4.9–5.2)
Metaplastic	10	6.2 (4.6–7.8)	10	4.9 (3.4–6.3)	10	2.1 (0.7–3.4)	10	2.5 (1.5–3.5)	10	5.2 (4.1–6.3)
Microcystic	10	6.1 (4.5–6.7)	10	7.9 (6.4–9.3)	10	1.4 (0.0–2.7)	9	2.6 (1.5–3.6)	10	6.0 (4.8–71)
Psammomatous	17	8.8 (7.6–10.0)	17	4.6 (3.5–5.8)	17	1.6 (0.6–2.6)	17	3.4 (2.6–4.1)	17	5.3 (4.4–6.1)
Secretory	35	9.8 (8.9–10.6)	35	9.6 (8.8–10.3)	35	3.7 (3.0–4.4)	35	2.7 (2.1–3.2)	35	4.3 (3.7–4.9)
Transitional	91	5.8 (5.3–6.3)	90	4.6 (4.1–5.0)	92	2.1 (1.7–2.5)	92	2.4 (2.1–2.8)	91	4.4 (4.0–4.7)
NOS	24	5.8 (4.8–6.8)	25	5.9 (5.0–6.8)	25	1.3 (0.4–2.1)	25	2.5 (1.8–3.1)	25	5.5 (4.8–6.2)
WHO II										
Atypical	84	5.1 (4.6–5.7)	86	6.4 (5.9–6.9)	86	2.4 (1.9–2.8)	85	2.4 (2.1–2.8)	86	4.6 (4.2–5.0)
Chordoid	10	7.2 (5.6–8.8)	10	8.3 (6.8–9.7)	10	2.0 (0.6–3.3)	10	2.1 (1.1–3.1)	9	5.2 (4.0–6.3)
Clear cell	0	-	0	-	0	-	0	-	0	-
WHO III										
Anaplastic	17	4.7 (3.5–5.9)	17	6.0 (4.9–7.1)	17	1.8 (0.8–2.8)	17	2.0 (1.2–2.8)	17	3.6 (2.7–4.4)
Papillary	0	-	0	-	0	-	0	-	0	-
Rhabdoid	6	4.6 (2.5–6.6)	6	4.0 (4.1–5.0)	6	2.7 (1.0–4.4)	6	1.9 (0.6–3.2)	6	2.5 (1.1–3.9)
Missing	19		15		13		11		9	
*p* Value (ANOVA)		< 0.0001*		< 0.0001*		0.0011*		0.0085*		< 0.0001*

Abbreviations: *SSTR* somatostatin receptor, *NOS* not otherwise specified, *CI* confidence interval, *WHO* World Health Organization, *ANOVA* analysis of variance; asterisk (*) presents statistically significant results

### General distribution of SSTR expression

**Fig. 1:**
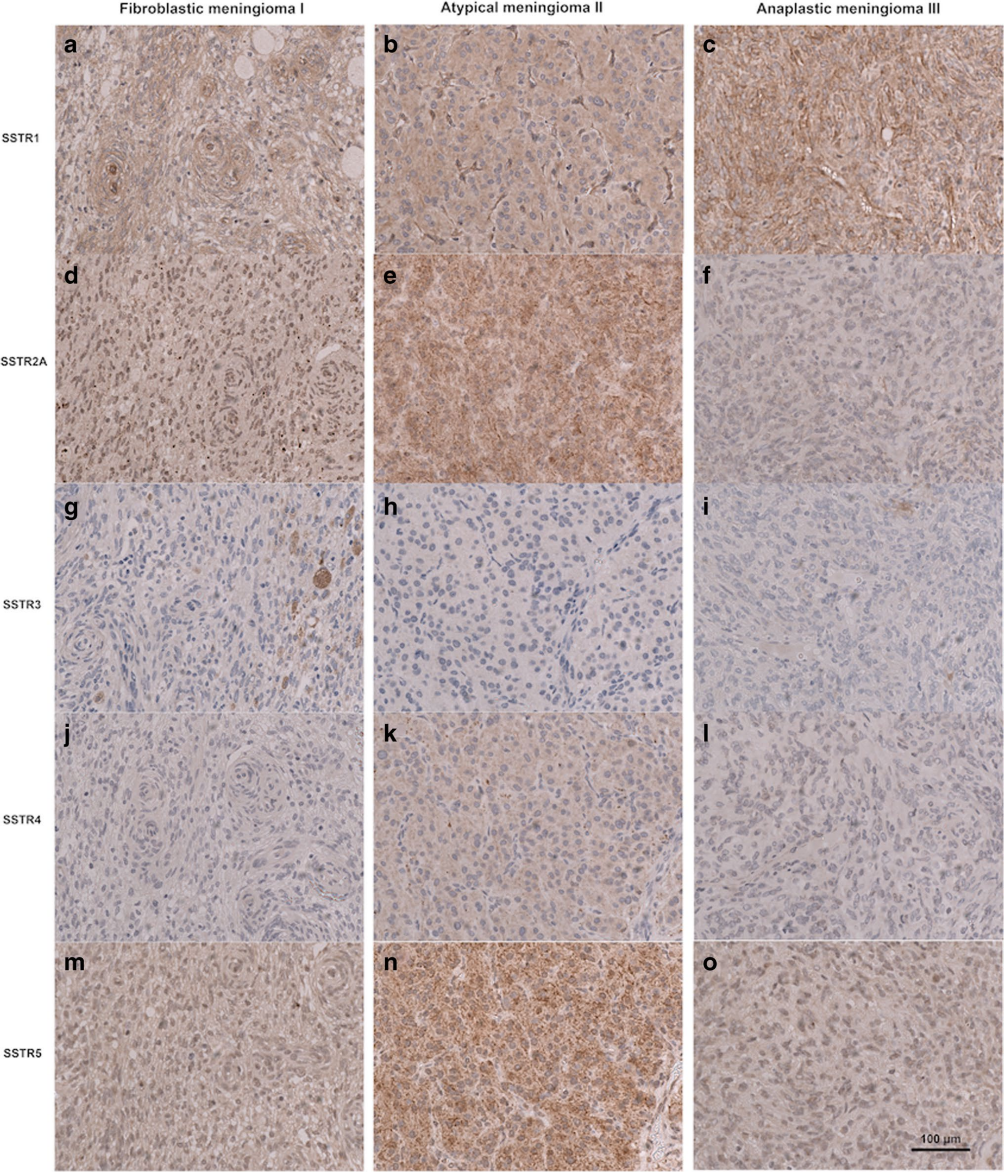
Examples of the immunohistochemical expression of somatostatin receptor 1 (**a–c**), 2A (**d–f**), 3 (**g–i**), 4 (**j–l**), and 5 (**m–o**) in three meningiomas of different WHO grades. Scale bar 100 µm

**Fig. 2:**
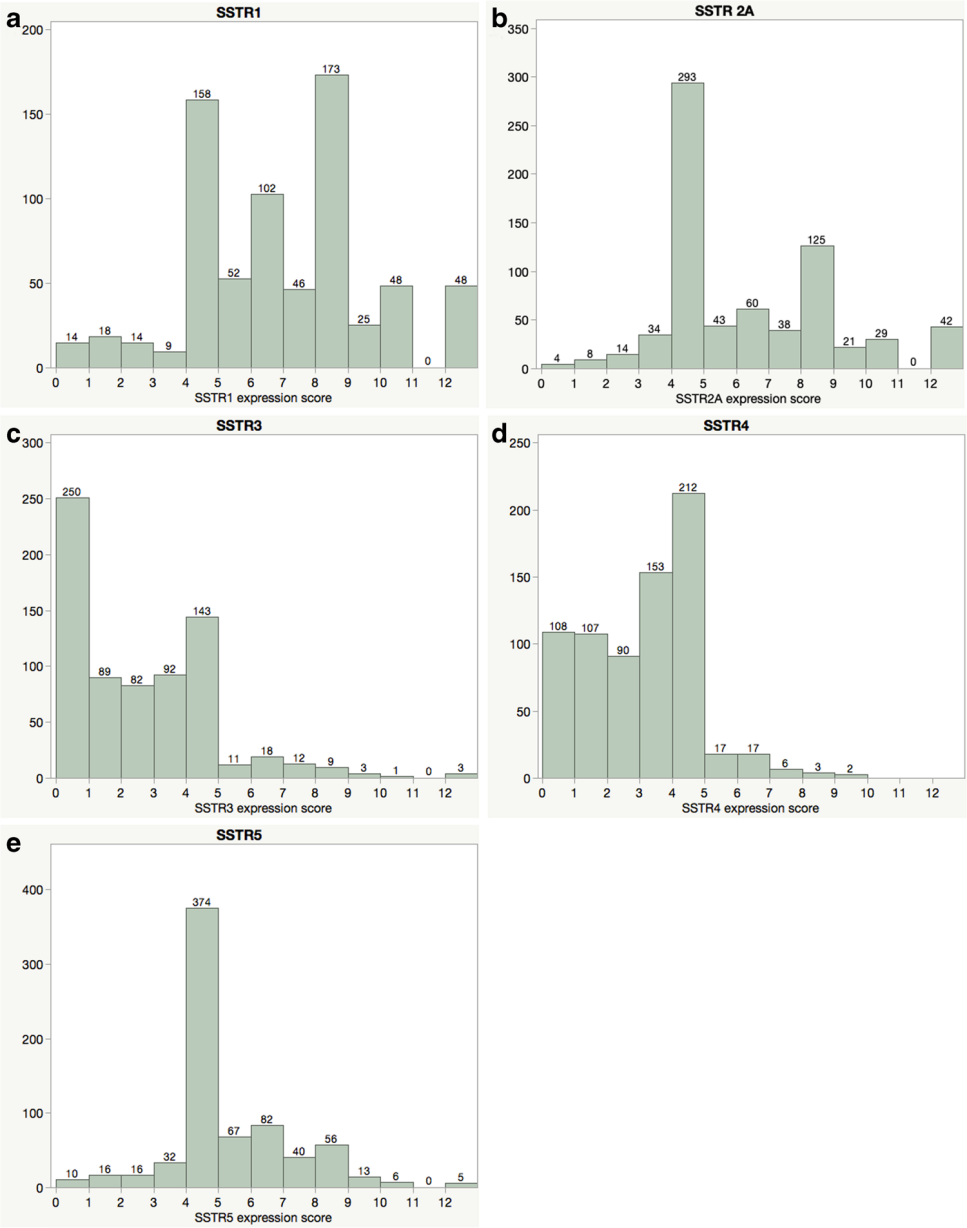
Distribution of the expression score of somatostatin receptors (**a**), 2A (**b**), 3 (**c**), 4 (**d**), and 5 (**e**). The *y*-axis presents the number of cases

After construction of tissue microarrays, microtomy, and staining, there were only a few cases lost for analysis. The reasons were insufficient tissue amount or staining for proper scoring due to tissue detachment in 19, 15, 13, 11, and 9 cases for SSTR1, 2A, 3, 4, and 5, respectively. All SSTR subtypes were expressed in meningiomas. Examples of the immunohistochemical staining in three cases is illustrated in Fig. 1. Cases with an expression score below 1 were graded as negative. Immunohistochemically negative cases were rare for SSTR1, 2A, and 5 (*n* = 14, 4, and 10 cases, respectively) but more common for SSTR3 and 4 (*n* = 250 and 108, respectively). The mean expression for SSTR1 and SSTR2A had the highest mean values (6.5 and 5.9, respectively), and the mean scores for SSTR3 and 4 were the lowest (2.2 and 2.7, respectively), while the mean expression score for SSTR5 was 4.8 (Fig. 2).

### Gender and age

A gender difference in expression score was only observed for SSTR1 with a slightly higher mean score in meningiomas of female patients (6.7 compared to 6.2, *p* = 0.0269). The age cutoffs determined by the CART analysis ranged from 34.5 to 72.5 years (Table 2) and with significantly higher expression scores for older patients for SSTR1, 2A, and 5 and vice versa for SSTR3 and 4 (Table 2).

### Tumor localization

**Fig. 3:**
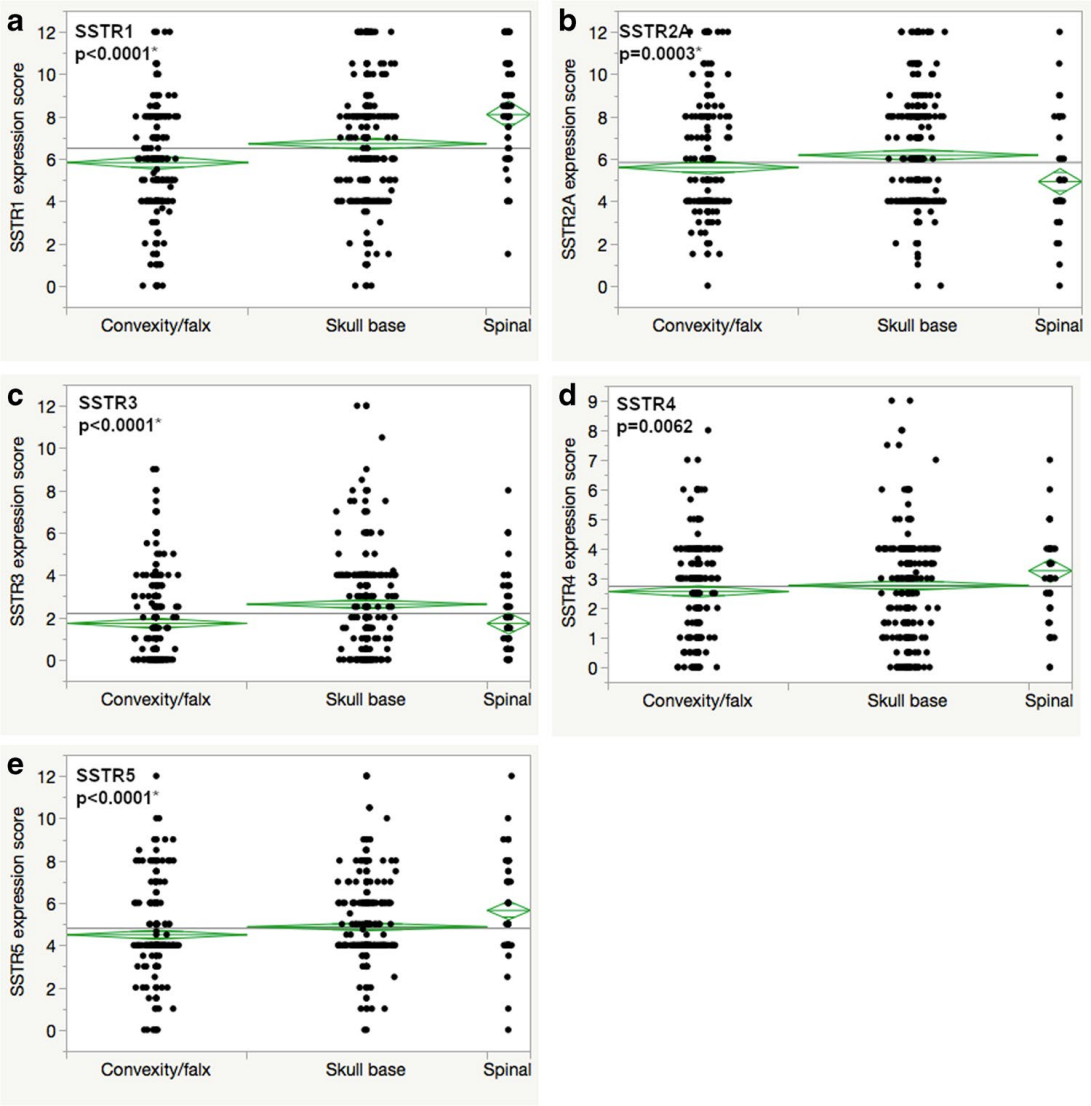
SSTR expression in different meningioma localizations (**a** SSTR1, **b** SSTR2A, **c** SSTR3, **d** SSTR4, **e** SSTR5); asterisk (*) presents statistically significant results

Somatostatin receptor expression scores varied between different tumor locations. Especially spinal meningiomas exhibited significantly higher expression scores for SSTR1, 4, and 5 and lower values for SSTR2A and 3, when compared to meningiomas of the skull base and the convexity/falx. This was most pronounced for SSTR1, where spinal meningiomas reached a mean score of 8.1, significantly higher compared to skull base and convexity/falx location (6.7 and 5.8, respectively, *p* < 0.0001). While the expression of SSTR4 was generally low in meningiomas when compared to other SSTRs (see Fig. 2), spinal tumor location showed the highest expression score (*p* = 0.0062). For SSTR5, the differences were more pronounced with 5.7 for spinal meningiomas, while skull base and convexity/falx locations reached a mean score of 4.5 and 4.9 (*p* < 0.0001). The highest expression score for SSTR2A was seen for skull base meningiomas with 6.2 followed by convexity/falx and spinal location (5.6 and 4.9, respectively, *p* = 0.0003). Skull base meningiomas also reached the highest score for SSTR3 (2.2), while spinal and convexity/falx tumors had similar low mean scores (1.7 each, *p* < 0.0001). For details, see Table 2 and Fig. 3.

### Recurrent and irradiated meningiomas

Meningiomas that were treated with radiotherapy before resection had significant lower scores for SSTR1 (5.4 compared to 6.6, *p* = 0.0007) and SSTR5 (4.3 compared 4.9, *p* = 0.0340), while expression of SSTR2A, 3, and 4 were similar to tumor tissue that did not receive radiotherapy. Recurrent meningiomas showed a significantly reduced expression for SSTR1 as well, when compared to primary meningiomas (5.5 vs. 6.7, *p* < 0.0001). For details, see Table 2 and Supplementary Figures S1 and S2.

### Neurofibromatosis type 2

**Fig. 4:**
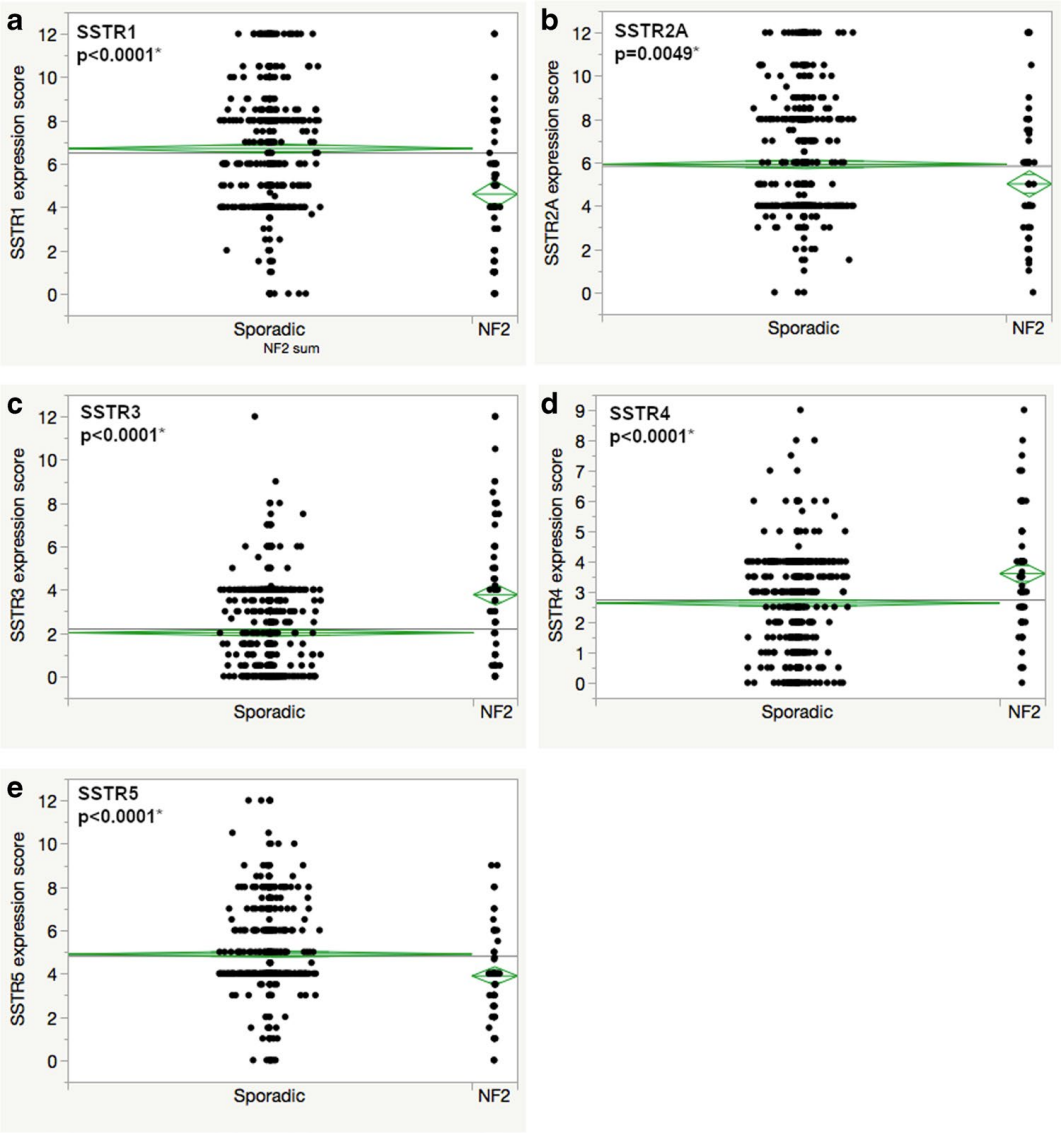
SSTR expression in meningiomas of patients suffering from neurofibromatosis type 2 compared to sporadic cases (**a** SSTR1, **b** SSTR2A, **c** SSTR3, **d** SSTR4, **e** SSTR5); asterisk (*) presents statistically significant results

Some of the largest differences of SSTR expression were observed when comparing NF2 and sporadic meningioma tissue. The expression scores of SSTR1, 2A, and 5 were significantly lower in meningiomas of NF2 patients. The largest difference was seen in SSTR1 with NF2 tumors reaching a mean score of 4.6 compared to 6.7 of sporadic meningiomas (*p* < 0.0001). On the contrary, the analysis of SSTR3 and 4 expression showed higher mean scores in NF2 meningiomas. For details, see Table 2 and Fig. 4.

### WHO grade and histologic subtype

**Fig. 5:**
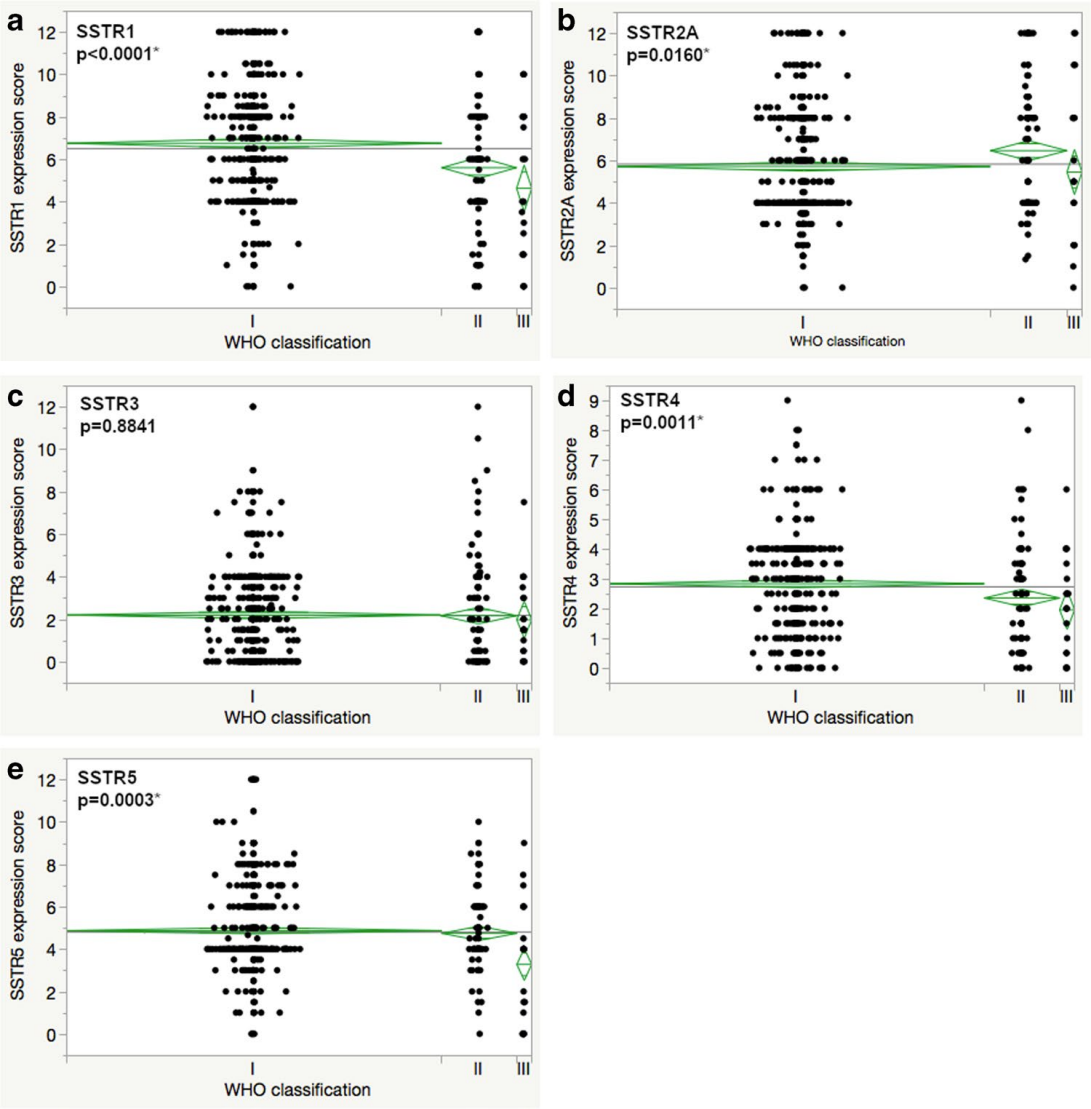
SSTR expression differences among meningioma WHO grades based on the current WHO classification of central nervous system tumors (**a** SSTR1, **b** SSTR2A, **c** SSTR3, **d** SSTR4, **e** SSTR5); asterisk (*) presents statistically significant results

Significant expression differences for WHO grades were seen for SSTR1, 2A, 4, and 5, while no differences were observed for SSTR3. For SSTR1 and 4, a gradual decrease of the mean expression score was seen between WHO grades I, II, and III (*p* < 0.0001 and *p* = 0.0011, respectively). The expression of SSTR2A was similar for grades I and III meningiomas, while grade II tumors revealed a higher mean expression score (*p* = 0.0160). Immunohistochemical staining for SSTR5 was scored similar for grade I and II tumors, and lower values were seen for grade III meningiomas (*p* = 0.0003). For details, see Table 2 and Fig. 5. Marked differences in expression scores between histologic subtypes were seen for all 5 SSTRs (Table 3). Several entities reached high expression scores for different markers. Secretory meningiomas were standing out with the highest mean expression score for SSTR1, 2A, and 3. Angiomatous meningiomas had also high scores for SSTR1 and 2 and the highest values for SSTR4 and 5. Among WHO grade II tumors, chordoid meningiomas had higher expression scores for SSTR1 and 2A compared to atypical meningiomas.

## Discussion

Our results show clear distinctions of somatostatin receptor expression in meningioma subgroups. Especially, SSTR1, 2A, and 5 show high expression rates. Among clinical subgroups, expression differences regarding tumor location, recurrent tumor, prior radiotherapy, neurofibromatosis type 2, and WHO grade were identified.

A deeper understanding of the distribution and role of somatostatin receptors in meningiomas is essential to further develop and refine a differentiated targeted application. Our study presents the largest analysis of somatostatin receptors in meningiomas. Prior studies have given first insights into the expression of SSTRs in small cohorts, ranging from 20 to 60 cases [2, 3, 11, 32, 36, 38], but most of them lacked significant subgroups that are potential recipients of a targeted peptide radio receptor therapy. Especially, patients suffering from recurrent or higher grade meningiomas as well as neurofibromatosis type 2, who often show multiple meningiomas, are in need of other treatment options. For example, one of the largest retrospective studies analyzed 60 meningiomas, including 11 grade II and 2 grade III tumors [38]. With over 100 meningiomas of higher WHO grade as well as large groups of tumors that received prior radiotherapy or have been resected from NF2 patients, we are able to describe the expression of somatostatin receptors in these highly relevant patient groups with marked differences that have the potential to be exploited for therapeutic purposes.

PRRT represents a promising therapy that has untapped potential in meningiomas. So far, only SSTR2A has been used as the target for PRRT, and its expression can be analyzed via PET imaging prior to treatment [18]. The intensity of the tracer uptake in the so-called DOTATAE/DOTATOC PET imaging can also predict the treatment response to SSTR2A-based PRRT [37] and outline residual or recurrent tumor tissue for further treatment planning [10]. With an integration of other somatostatin receptors into SSTR-PET imaging, this diagnostic tool could be further refined. The efficacy of PRRT has been shown in several studies with mostly small cohorts ranging from 10 to 20 patients [13, 19]. Higher immunohistochemical expression of SSTR2A in meningioma tissue prior to PRRT was associated with longer progression-free survival in a small retrospective analysis of 18 cases [37]. Although these retrospective studies are small and are composed of mostly recurrent and treatment resistant, higher grade meningiomas, treatment efficacy was seen if high receptor expression was detected. With the new insights of our study, a more specific and patient-tailored treatment according to a multi receptor expression is possible.

For example, our data clearly show lower expression rates for SSTR1, 2A, and 5 in meningiomas from NF2 patients and increased scores for SSTR3 and 4. If these differences are associated with a lower efficacy of PRRT, which is currently administered SSTR2-specific, remains unclear. But it is possible that a substance with a multi receptor affinity may result in better therapy response. A similar argument can be made for spinal meningiomas that showed the lowest SSTR2A expression compared to other meningioma localizations but the highest for SSTR1, 4, and 5. Again, our results suggest that other PRRT substances could be more ideal for this tumor subgroup. It should be kept in mind that especially spinal meningiomas are less likely to recur or develop an aggressive behavior [14], thus making them not the classic subgroup for PRRT. Furthermore, skull base meningiomas are challenging to operate and are thus more likely to be subtotally resected. Meningiomas of this location reached the highest SSTR2A expression score, but also possessed high expressions of SSTR1 and 5, underlining the potential of a multi receptor target for PRRT.

Regarding histology, SSTR expression showed large variations. Secretory and angiomatous were among the highest SSTR expressing meningioma subtypes, making these entities especially interesting targets for PRRT. Furthermore, these entities are known for non-NF2 molecular alterations [8, 33]. However, meningiomas of higher WHO grade are more likely to reach a point where alternative therapy options like PRRT can be very helpful. Unfortunately, WHO grade III tumors in our cohort showed the lowest expression scores for all SSTRs. It can be argued that lower SSTR expression with higher WHO grade may be due to a dedifferentiation of meningiomas as has been suggested in regard to the loss of the expression of hormone receptors is higher grade meningiomas [20, 22]. Of course, this does not automatically suggest that these tumors should be precluded from PRRT. It rather stresses the need to develop multi affinity substances. Even for grade II meningiomas that show a high expression of SSTR2A, good expression was also seen for SSTR1 and 5, implying that PRRT targeting all SSTRs, but especially SSTR1, 2A, and 5, may be beneficial in delivering radiation to tumor cells more sufficiently.

Substances that target multiple somatostatin receptors with high affinity have been developed and evaluated for the treatment of neuroendocrine tumors and acromegaly [28]. Especially the new generation substance pasireotide has a high affinity to multiple SSTR receptors with a reported 39-fold affinity to SSTR5 compared to octreotide [28]. Furthermore, administering the long-acting somatostatin analog octreotide by itself has been shown to be efficacious in a subgroup of recurrent meningiomas with a partial radiographic response in 5 of 16 patients after 6 months [7]. A phase II clinical trial has demonstrated a positive response in a group of 20 recurrent meningiomas after administration of octreotide combined with the mTOR inhibitor everolimus [17]. However, the superior antiproliferative activity of pasireotide and especially decreased cell viability in combination with everolimus has been shown in vitro [16]. Overall, there is a lot of potential in the further development and refinement of PRRT to optimize the efficacy of this highly promising treatment approach. Our data demonstrate how differences in clinical subgroups could be utilized for a more tailored PRRT.

Furthermore, it is of interest to assess the response to PRRT depending on the immunohistochemical receptor expression. Although there are defined cutoffs for SSTR2-based PET imaging [37], it remains unclear what degree of immunohistochemical receptor expression may be sufficient for an efficacious PRRT. In addition to that, it is still unknown how the receptor expression may behave after radiotherapy or PRRT. These are questions we plan to address with future projects.

The main limitation of the presented study is its retrospective nature. Due to the expertise in skull base surgery and neurofibromatosis type 2, the cohort includes more meningiomas of the skull base as well as recurrent and NF2 associated tumors. Furthermore, the differences in SSTR expression are in some cases quite small, and it is unclear if this is associated with a difference in biology or response to PRRT. However, the goal of this study was the description of SSTR distribution and the comparison of clinical subgroups. The biological relevance of the different immunohistochemical SSTR expression levels will be the subject of further research efforts.

## Conclusion

The expression of somatostatin receptors 1, 2A, 3, 4, and 5 in meningiomas shows differences between relevant clinical subgroups, especially recurrent or radiated tumors and meningiomas resected from NF2 patients. Overall, high expressions of SSTR1, 2A, and 5 were seen. Thus, a broader receptor affinity of substances used for peptide radioreceptor therapy has the potential to improve the treatment delivery in meningioma tissue.

## Supplementary Information

Below is the link to the electronic supplementary material.

Supplementary Fig. S1 SSTR expression in meningiomas that were treated with prior radiotherapy compared to untreated tumors (A: SSTR1, B: SSTR2A, C: SSTR3, D: SSTR4, E: SSTR5), asterisk(*) presents statistically significant results (PNG 114 KB)

Supplementary Fig. S2 SSTR expression in primary and recurrent meningiomas (A: SSTR1, B: SSTR2A, C: SSTR3, D: SSTR4, E: SSTR5), asterisk(*) presents statistically significant results (PNG 115 KB)

## Data Availability

The dataset is available upon reasonable request.

## Code availability

No applicable.
