# Supplementary figures and images for: Differences in the expression of SSTR1–5 in meningiomas and its therapeutic potential
Source: Neurosurg Rev. 2021 Apr 26;45(1):467–78. doi: 10.1007/s10143-021-01552-y (PMC8827401; doi:10.1007/s10143-021-01552-y)

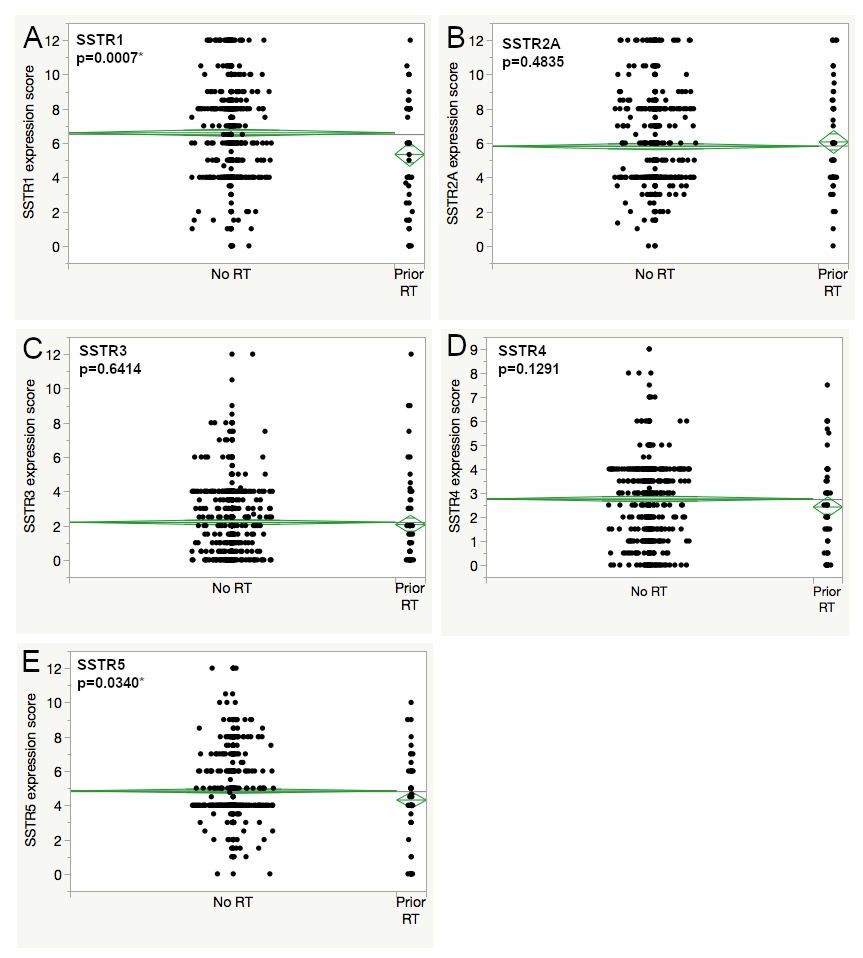

Supplement: Supplementary file 1 — SSTR expression in meningiomas that were treated with prior radiotherapy compared to untreated tumors (A: SSTR1, B: SSTR2A, C: SSTR3, D: SSTR4, E: SSTR5), asterisk(*) presents statistically significant results (PNG 114 KB) [file 10143_2021_1552_MOESM1_ESM.png]

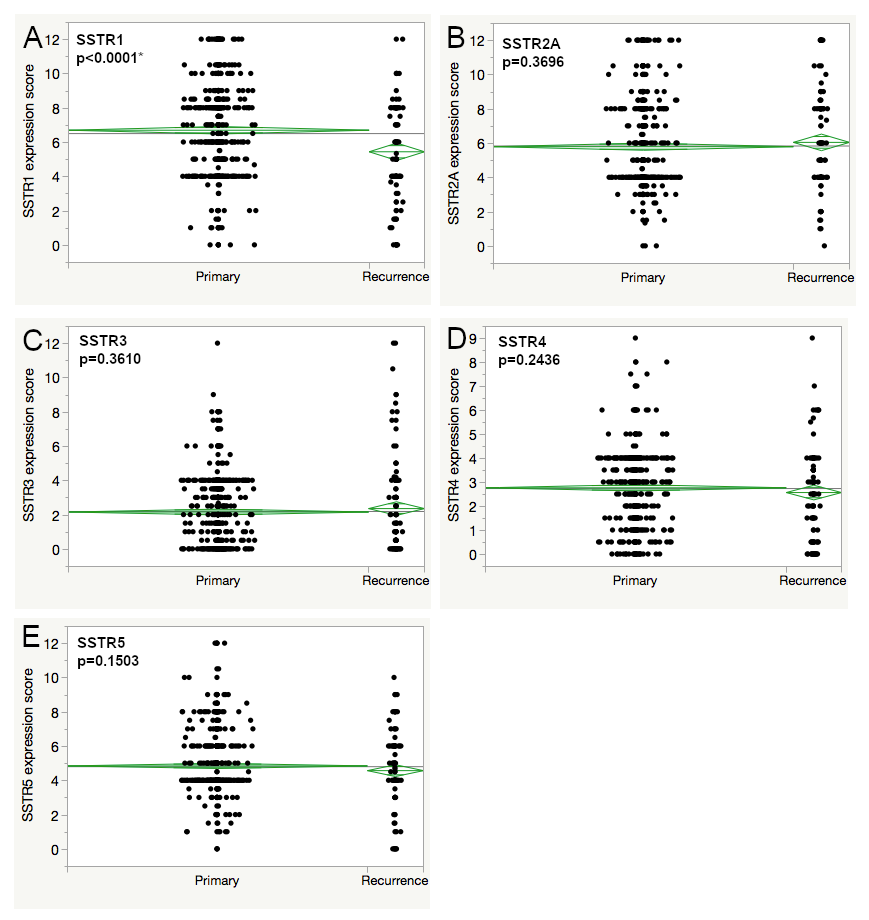

Supplement: Supplementary file 2 — SSTR expression in primary and recurrent meningiomas (A: SSTR1, B: SSTR2A, C: SSTR3, D: SSTR4, E: SSTR5), asterisk(*) presents statistically significant results (PNG 115 KB) [file 10143_2021_1552_MOESM2_ESM.png]
